# Association of smoking behavior and time to first cigarette with all-cause and cause-specific mortality: A cohort analysis from the NHANES 2001–2018

**DOI:** 10.18332/tid/215944

**Published:** 2026-02-19

**Authors:** Naiyue Bao, Jiacan Wu, Zhuo Li, Fenglin Qi, Guanghong Tao, Chengcheng Li, Hua Xiao

**Affiliations:** 1 Department of Cardiology, The First Affiliated Hospital of Chongqing Medical University, Chongqing, China

**Keywords:** all-cause mortality, cause-specific mortality, time to first morning cigarette, nicotine dependence, NHANES

## Abstract

**INTRODUCTION:**

Smoking is a major risk factor for death, but the impact of behavioral patterns such as time to first cigarette (TTFC) on this risk has been little studied in the US population. To address this, we investigated the association between smoking status and TTFC with all-cause, cardiovascular disease, and cancer mortality based on a national sample.

**METHODS:**

We conducted a pooled secondary data analysis of the National Health and Nutrition Examination Survey data from the United States, covering nine continuous cycles between 2001 and 2018. The initial pooled sample comprised 91355 participants. After applying a series of exclusion criteria, the final analytical cohort consisted of 39084 adult subjects. TTFC was investigated by means of a touch-screen questionnaire. Outcomes included all-cause mortality, cardiovascular disease mortality, and malignancy-related mortality. Mortality information was obtained from the National Death Index (NDI) using death certificates (https://www.cdc.gov/nchs/linked-data/mortality-files/?CDC_AAref_Val=https://www.cdc.gov/nchs/data-linkage/mortality-public.htm).

**RESULTS:**

After full adjustment, former smokers had a significantly elevated risk of mortality compared to never smokers. A U-shaped association was observed between TTFC and both all-cause and CVD mortality. For all-cause mortality, the risk was highest in smokers with TTFC <30 min (hazard ratio, HR=4.47; 95% CI: 2.23–8.98, p<0.001). Similarly, for CVD mortality, the highest risk was also found in the TTFC <30 min group (HR=5.53; 95% CI: 2.32–13.14, p<0.001). In contrast, TTFC showed an inverse relationship with cancer mortality, with the risk being highest for TTFC <30 min (HR=3.23; 95% CI: 1.25–8.31, p=0.020) and decreasing with a later TTFC.

**CONCLUSIONS:**

Former and current smokers showed elevated all-cause, CVD, and cancer mortality risks versus never smokers. TTFC exhibited a U-shaped association with all-cause and CVD mortality, but not with cancer mortality.

**ABBREVIATIONS:**

TTFC: time to first cigarette, NHANES: National Health and Nutrition Examination Survey, NDI: National Death Index, NCHS: National Center for Health Statistics, CDC: Centers for Disease Control and Prevention, HEI-2020: Healthy Eating Index, GED: General Educational Development, PIR: poverty-to-income ratio, BMI: body mass index, CVD: cardiovascular disease, TC: total cholesterol, ALT: alanine aminotransferase, AST: aspartate aminotransferase, HbA1C: glycated hemoglobin, WTDR1D: dietary one-day sample weight

## INTRODUCTION

Globally, smoking remains the leading cause of preventable disease and premature death, responsible for 435000 annual US deaths^1^. Smokers live on average <10 years compared to never smokers. Quitting at ages 30, 40, 50, and 60 years gains an average of 10, 9, 6, and 3 years of life expectancy, respectively, underscoring cessation as a key intervention^2^. However, unaided quit attempts face high relapse rates, with 80% resuming smoking within one month and only about 3% achieving long-term cessation without support^3^. The establishment of quantifiable risk indicators would be valuable to identify at-risk populations, given the prevalence of intractable dependence.

TTFC is significantly associated with the degree of nicotine dependence^4^. More importantly, almost all studies assessing the association of TTFC with biomarkers of cigarette exposure have found that exposure levels are negatively associated with TTFC regardless of the biomarkers used (e.g. cotinine^5^, carboxyhemoglobin, and carbon monoxide^6^). The consistency of these findings confirms that TTFC can be used as a reliable behavioral indicator of tobacco exposure^7^. Therefore, clinicians can use TTFC as a screening tool to assess nicotine dependence. Several studies have demonstrated that short TTFC predicts all-cause mortality and risk of cardiovascular disease death^8^. This association is of particular interest because all-cause mortality is a fundamental indicator of overall population health^9,10^. However, there is a lack of dose-response relationship studies and direct comparison data with previous smokers, both of which are clinically important.

This investigation aimed to identify individuals at high mortality risk using the TTFC and, by examining its association with all-cause and cause-specific mortality, provide an evidence base for tailored clinical smoking cessation interventions.

## METHODS

### Study design and participants

This study is a retrospective analysis of secondary data from the cross-sectional National Health and Nutrition Examination Survey (NHANES), which is administered by the National Center for Health Statistics (NCHS). NHANES employs a sophisticated multistage sampling design to obtain nationally representative samples of the US population using stratification and probability sampling methods. This database primarily serves to evaluate both health parameters and nutritional metrics across the US population thereby encompassing all age demographics^11^. The NCHS authorized all research procedures, and written informed consent was secured from every enrolled individual. Consequently, this investigation strictly complied with established ethical standards thus eliminating the necessity for supplementary ethics evaluations. From an initial pooled sample of 91355 individuals, we excluded 52271 participants for the following reasons: no smoking status (n=40307), missing dietary weights (n=5779), absent mortality follow-up (n=81) or cause-specific mortality data (n=2703), pregnancy (n=1137), death within 12 months of follow-up (n=259), and missing exposure data (n=2703). Consequently, 39084 participants were included in the final analysis.

### Exposure

Data on smoking status and the TTFC after waking up were collected using a baseline touchscreen questionnaire. The smoking status was determined using data from the NHANES Cigarette Use Questionnaire. Participants were categorized into five groups based on smoking behavior: non-smokers and former smokers, and three groups of smokers based on the TTFC: <30 minutes, 30–60 minutes, and >60 min (Supplementary file Table 1)^12^.

### Mortality

Mortality information was obtained from the NDI using death certificates (https://www.cdc.gov/nchs/linked-data/mortality-files/?CDC_AAref_Val=https://www.cdc.gov/nchs/data-linkage/mortality-public.htm). Therefore, before 31 December 2019, the respective mortality information for each participant was determined by connecting to the NDI. The follow-up time for Kaplan-Meier and Cox proportional hazards models was calculated in months, spanning from the interview date to the date of either death or the study endpoint. Disease-specific mortality was categorized pursuant to ICD-10 specifications. Cardiovascular disease fatalities were delineated under codes I00-I09, I11, I13, and I20-I51 per standardized classification protocols. Cancer mortality rates were classified using codes C00–C97.

### Covariates

In this study, we collected data on sociodemographic characteristics, health status characteristics, anthropometry, and laboratory test results obtained through computer-assisted personal interviews. Sociodemographic characteristics include age (years), sex (male, female), race (Mexican American, non-Hispanic White, non-Hispanic Black, other Hispanic, and other races), education level (lower than high school, high school or General Educational Development (GED), and higher than high school), marital status (married/living with a partner, widowed/divorced/separated, and never married), and the household income was represented by the ratio of household income to poverty (PIR) and categorized as low (PIR <1.3), medium (1.3 ≤ PIR <3.5), and high (PIR ≥3.5). Health status characteristics include drinking status (light drinking/moderate drinking/heavy drinking), diabetes (yes, no), hypertension (yes, no), history of CVD (yes, no), and history of cancer (yes, no). Specifically, alcohol consumption patterns were categorized pursuant to National Institute on Alcohol Abuse and Alcoholism criteria as follows: light (≤1 daily beverage for females or ≤2 for males); moderate (2–3 daily beverages for females or 3–4 for males); and heavy (>3 daily beverages for females or >4 for males)^13^. History of hypertension, diabetes, CVD, and cancer was self-reported by participants. Body composition was assessed primarily via body mass index (BMI, kg/m²), stratification with defined thresholds: lean (<18.5), normal (18.5–24.9), overweight (25.0–29.9), and obese (≥30.0)^14^. Dietary quality was quantified using the Healthy Eating Index-2020 (HEI-2020). This scale, which ranges from 0 to 100, evaluates the intake of various food components (e.g. fruits, vegetables, whole grains, added sugars) based on 24-hour dietary recall data. A higher score reflects closer adherence to dietary recommendations and thus a healthier diet. Physical activity was classified into low, moderate, and vigorous exercise^15^. Laboratory tests include total cholesterol (TC, mg/dL), alanine aminotransferase (ALT, U/L), aspartate aminotransferase (AST, U/L), glycated hemoglobin (HbA1C, %), blood glucose (mg/dL), white blood cell count (10^9^/L), and neutrophil count (10^9^/L). Additionally, smoking-related variables were included as confounders to assess the independent risk of TTFC. These comprised smoking pack-years (calculated as the number of packs smoked per day multiplied by the number of years smoked), smoking duration (years), number of cigarettes smoked per day, and age at smoking initiation (years). Detailed explanations of these variables can be found on the official NHANES web site.

### Statistical analysis

Multiple imputation by chained equations was employed to handle missing data. We assumed that the data were missing at random, which implies that the probability of a value being missing may depend on the observed data but not on the unobserved data. For missing data in the covariates, we performed multiple imputation using chained equations (using the mice package in R software, version 4.4.0). A random seed was set to 123, and 5 imputed datasets were generated, each undergoing 10 iterations. Model convergence was verified by plotting trace plots of the imputed variables during the iterations. Frequencies (n) and percentages (%) are used to represent categorical variables, whereas continuous variables are expressed as means with standard deviations (SD). To assess differences between groups, normally distributed data were analyzed using analysis of variance, and categorical variables were tested using chi-squared tests. Kaplan-Meier curves estimated survival probabilities for all-cause, cardiovascular, and cancer mortality by smoking status without adjustment, with group differences assessed using log-rank tests. Adjusted hazard ratios (AHR) and 95% confidence interval (CI) were derived from Cox models to evaluate associations between smoking status and mortality outcomes, with covariates added sequentially. Following the principles of strengthening the reporting of epidemiological observational studies, three models were established: Model 1 included age and sex; Model 2 added demographic factors, health behaviors, clinical comorbidities, and laboratory biomarkers; Model 3 further incorporated smoking-related variables. The proportional hazards assumption was verified using Schoenfeld residuals. Our initial Cox regression analyses indicated that the relationship between TTFC and mortality might not be strictly linear. We therefore explored the possibility of a nonlinear relationship by fitting extended models that included a quadratic polynomial term for TTFC, which provided a significantly better fit to the data. This study uses the Fine-Gray competing risks model to assess the specific impact of smoking status on cardiovascular and cancer mortality, addressing the competing risks posed by non-target causes of death (e.g. cancer mortality when analyzing cardiovascular mortality, and vice versa). All-cause mortality is evaluated using the conventional Cox proportional hazards model.

In addition, we conducted stratified analyses based on age, sex, race, PIR, education level, alcohol consumption intensity, HEI2020, physical activity, hypertension, diabetes, and high cholesterol to assess whether the associations between smoking status and all-cause and cause-specific mortality were influenced by these variables^16^. The same Cox model was analyzed with the inclusion of an interaction term. P-values for the interactions were assessed using interaction terms and likelihood ratio tests. Interaction p-values >0.05 were considered to indicate robust results across different strata.

Furthermore, joint analyses of smoking-related variables and different smoking status were conducted to examine their associations with all-cause and cause-specific mortality.

To further validate the results, we conducted the following two sensitivity analyses. First, we excluded participants who died within two years of follow-up. Second, we excluded participants with missing covariate data. Nine cycles of the NHANES were integrated and analyzed using WTDRD1 sample weights, accounting for stratification and clustering owing to the complex sampling design. Data were analyzed using R software (version 4.4.0) and statistical significance was set at p<0.05.

## RESULTS

### Basic characteristics

**Figure 1:**
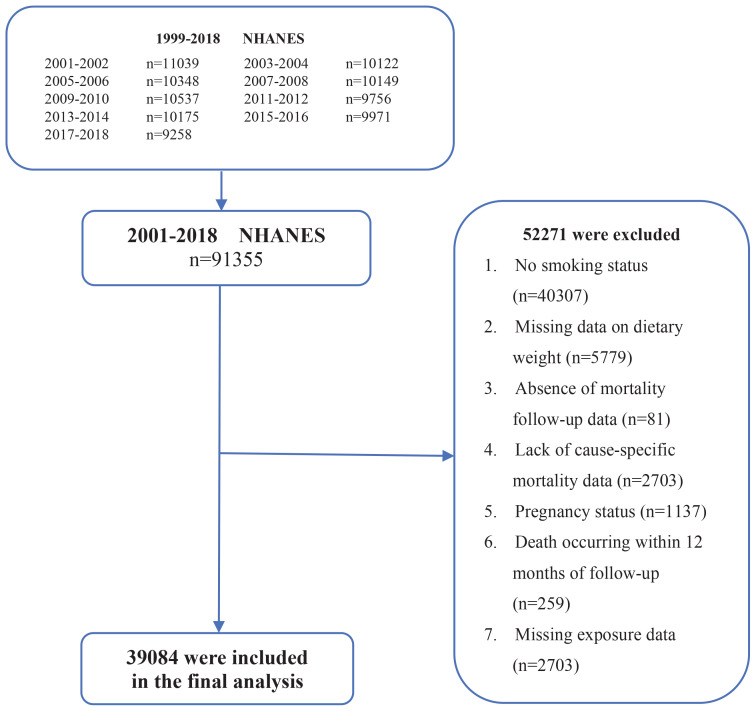
Flowchart of participant selection in a prospective cohort study on smoking and mortality using the US National Health and Nutrition Examination Survey (NHANES) data, 2001–2018

This cohort study ultimately included 39084 participants from nine rounds of NHANES surveys (2001–2018). The participant selection process is detailed in Figure 1, which shows that a total of 52271 individuals were excluded due to missing data on key variables. The mean age of the included participants varied significantly across smoking status groups, ranging from 42 years in the TTFC >60 min group, to 57 years in the former smoker group. Individuals with a shorter TTFC in the morning were predominantly men and non-Hispanic Whites with a lower level of education, unhealthy diets, heavy alcohol consumption, elevated inflammatory marker levels, and impaired glucose metabolism. These individuals also tended to smoke more heavily (Table 1). All these observed associations were statistically significant.

**Table 1:** Baseline characteristics of study participants by smoking status, National Health and Nutrition Examination Survey (NHANES), United States, 2001–2018 (N=39084)

*Characteristics*	*Non-smoker* *n (%)*	*Former* *smoker* *n (%)*	*Smoker TTFC (min)*			*p*
			*<30* *n (%)*	*30–60* *n (%)*	*>60* *n (%)*	
**Total,** n	22846	9618	4110	1333	1177	
**Age** (years), mean ± SD	46 ± 18	57 ± 16	45 ± 14	43 ± 16	42 ± 15	<0.001**^a^**
**Gender**						<0.001**^b^**
Male	9452 (41.4)	5849 (60.8)	2305 (56.1)	734 (55.1)	665 (56.5)	
Female	13394 (58.6)	3769 (39.2)	1805 (43.9)	599 (44.9)	512 (43.5)	
**Ethnicity**						<0.001**^b^**
Non-Hispanic White	8593 (37.6)	5114 (53.2)	2376 (57.8)	685 (51.4)	444 (37.7)	
Non-Hispanic Black	5052 (22.1)	1575 (16.4)	1040 (25.3)	347 (26.0)	284 (24.1)	
Mexican American	4300 (18.8)	1505 (15.6)	243 (5.9)	129 (9.7)	269 (22.9)	
Other race/multi-racial	2682 (11.7)	640 (6.7)	250 (6.1)	97 (7.3)	83 (7.1)	
Other Hispanic	2219 (9.7)	784 (8.2)	201 (4.9)	75 (5.6)	97 (8.2)	
**Education level**						<0.001**^b^**
High school or higher	13057 (57.2)	4967 (51.6)	1416 (34.5)	568 (42.6)	447 (38.0)	
High school or GED	4736 (20.7)	2246 (23.4)	1322 (32.2)	415 (31.1)	326 (27.7)	
High school or lower	5053 (22.1)	2405 (25.0)	1372 (33.4)	350 (26.3)	404 (34.3)	
**Marital status**						<0.001**^b^**
Widowed/divorced/separated	4245 (18.6)	2316 (24.1)	1089 (26.5)	316 (23.7)	260 (22.1)	
Never married	4657 (20.4)	930 (9.7)	866 (21.1)	319 (23.9)	290 (24.6)	
Married/living with partner	13944 (61.0)	6372 (66.3)	2155 (52.4)	698 (52.4)	627 (53.3)	
**PIR**						<0.001**^b^**
Moderate PIR (1.3≤ PIR <3.5)	8584 (37.6)	3804 (39.6)	1457 (35.5)	524 (39.3)	477 (40.5)	
High PIR (PIR ≥3.5)	7823 (34.2)	3401 (35.4)	654 (15.9)	281 (21.1)	253 (21.5)	
Low PIR (PIR <1.3)	6439 (28.2)	2413 (25.1)	1999 (48.6)	528 (39.6)	447 (38.0)	
**HEI2020_ALL** (score range: 0–100), mean ± SD	52 ± 12	53 ± 12	45 ± 10	47 ± 10	47 ± 11	<0.001**^b^**
**BMI** (kg/m^2^)						<0.001**^b^**
Obese (≥30.0)	8616 (37.7)	3911 (40.7)	1277 (31.1)	438 (32.9)	399 (33.9)	
Overweight (25.0–29.9)	7470 (32.7)	3545 (36.9)	1236 (30.1)	415 (31.1)	393 (33.4)	
Normal weight (18.5–24.9)	6433 (28.2)	2082 (21.6)	1459 (35.5)	443 (33.2)	354 (30.1)	
Underweight (<18.5)	327 (1.4)	80 (0.8)	138 (3.4)	37 (2.8)	31 (2.6)	
**Physical activity intensity**						<0.001^b^
Moderate	16511 (72.3)	6976 (72.5)	2858 (69.5)	1001 (75.1)	846 (71.9)	
Light	5275 (23.1)	2288 (23.8)	1079 (26.3)	274 (20.6)	275 (23.4)	
Vigorous	1060 (4.6)	354 (3.7)	173 (4.2)	58 (4.4)	56 (4.8)	
**Cancer**						<0.001^b^
No	21200 (92.8)	8237 (85.6)	3791 (92.2)	1255 (94.1)	1122 (95.3)	
Yes	1646 (7.2)	1381 (14.4)	319 (7.8)	78 (5.9)	55 (4.7)	
**Diabetes**						<0.001^b^
No	19552 (85.6)	7470 (77.7)	3547 (86.3)	1180 (88.5)	1048 (89.0)	
Yes	3294 (14.4)	2148 (22.3)	563 (13.7)	153 (11.5)	129 (11.0)	
**Hypertension**						<0.001^b^
No	14656 (64.2)	4761 (49.5)	2528 (61.5)	887 (66.5)	802 (68.1)	
Yes	8190 (35.8)	4857 (50.5)	1582 (38.5)	446 (33.5)	375 (31.9)	
**Having a family history of heart disease**						<0.001^b^
No	21218 (92.9)	8005 (83.2)	3609 (87.8)	1215 (91.1)	1083 (92.0)	
Yes	1628 (7.1)	1613 (16.8)	501 (12.2)	118 (8.9)	94 (8.0)	
**Alcohol drinking**						<0.001^b^
Light	16323 (71.4)	6596 (68.6)	2002 (48.7)	606 (45.5)	481 (40.9)	
Moderate	3242 (14.2)	1478 (15.4)	633 (15.4)	253 (19.0)	221 (18.8)	
Heavy	3281 (14.4)	1544 (16.1)	1475 (35.9)	474 (35.6)	475 (40.4)	
**Smoking status**						
Age started smoking (years), mean ± SD	-	17 ± 6	17 ± 5	18 ± 6	19 ± 7	<0.001^a^
Cigarettes/day, mean ± SD	-	0.45 ± 0.57	0.84 ± 0.57	0.59 ± 0.47	0.45 ± 0.51	<0.001^a^
Pack-years, mean ± SD	-	19 ± 25	25 ± 22	15 ± 16	10 ± 13	<0.001^a^
Duration of smoking (years), mean ± SD	-	40 ± 17	28 ± 15	25 ± 16	23 ± 15	<0.001^a^
**Health status**						
Total cholesterol (mg/dL), mean ± SD	4.99 ± 1.05	5.07 ± 1.12	5.08 ± 1.17	5.01 ± 1.08	5.02 ± 1.05	<0.001^a^
Alanine aminotransferase (U/L), mean ± SD	25 ± 18	26 ± 20	24 ± 17	26 ± 25	28 ± 61	<0.001^a^
Aspartate aminotransferase (U/L), mean ±						
SD	25 ± 16	26 ± 16	25 ± 15	26 ± 20	27 ± 51	<0.001^a^
Blood glucose (mg/dL), mean ± SD	100 ± 36	106 ± 40	99 ± 35	97 ± 30	98 ± 31	<0.001^a^
Glycated hemoglobin (%), mean ± SD	5.68 ± 1.05	5.85 ± 1.09	5.69 ± 1.02	5.61 ± 0.89	5.62 ± 0.94	<0.001^a^
White blood cell count (109/L), mean ± SD	6.99 ± 3.50	7.07 ± 2.41	8.34 ± 2.52	8.11 ± 2.31	7.66 ± 2.11	<0.001^a^
Neutrophil count (109/L), mean ± SD	57 ± 9	58 ± 10	59 ± 10	59 ± 9	58 ± 9	<0.001^a^
Follow-up time (months), mean ± SD	107 ± 60	107 ± 60	114 ± 59	117 ± 61	132 ± 57	<0.001^a^
**Mortality**						
All-cause						<0.001^b^
No	21524 (94.2)	8300 (86.3)	3693 (89.9)	1238 (92.9)	1095 (93.0)	
Yes	1322 (5.8)	1318 (13.7)	417 (10.1)	95 (7.1)	82 (7.0)	
**CVD**						<0.001**^b^**
No	21961 (96.1)	8836 (91.9)	3905 (95.0)	1285 (96.4)	1130 (96.0)	
Yes	885 (3.9)	782 (8.1)	205 (5.0)	48 (3.6)	47 (4.0)	
**Cancer**						<0.001**^b^**
No	22409 (98.1)	9082 (94.4)	3898 (94.8)	1286 (96.5)	1142 (97.0)	
Yes	437 (1.9)	536 (5.6)	212 (5.2)	47 (3.5)	35 (3.0)	

a One-way analysis of means.

b Pearson’s chi-squared test. TTFC: time to first cigarette after waking. PIR: poverty-income ratio. GED: General Educational Development (high school equivalence). BMI: body mass index. CVD: cardiovascular disease. HEI2020: Healthy Eating Index-2020.

### Association between different smoking statuses and all-cause mortality

In Model 1, former smokers showed a significantly higher hazard ratio than non-smokers (adjusted hazard ratio, AHR=1.29; 95% CI: 1.08–1.37, p<0.001), while current smokers exhibited the highest risk. A U-shaped association was observed between TTFC and all-cause mortality, with AHR of 3.37 (95% CI: 1.96–2.80, p<0.001) for TTFC <30 min, 1.70 (95% CI: 1.09–1.78, p<0.001) for 30–60 min, and 1.96 (95% CI: 1.10–2.29, p<0.001) for >60 min (Table 2).

**Table 2:** Hazard ratios for the association between smoking status and all-cause mortality among US adults, National Health and Nutrition Examination Survey (NHANES), United States, 2001–2018 (N=39084)

*Smoking status*	*Model 1*			*Model 2*			*Model 3*		
	*AHR*	*95% CI*	*p*	*AHR*	*95% CI*	*p*	*AHR*	*95% CI*	*p*
**Non-smoker**	1	-	-	1	-	-	1	-	-
**Former smoker**	1.29	1.08–1.37	<0.001	1.22	1.08–1.37	0.001	2.58	1.24–5.38	0.012
**Smoker TTFC** (min)									
<30	3.37	1.96–2.80	<0.001	2.35	1.96–2.80	<0.001	4.47	2.23–8.98	<0.001
30–60	1.70	1.09–1.78	<0.001	1.40	1.10–1.78	0.008	2.84	1.36–5.94	0.006
>60	1.96	1.10–2.29	0.010	1.59	1.20–2.29	0.014	3.34	1.62–6.86	0.001

The non-smoker group served as the reference category. AHR: adjusted hazard ratio. Model 1: adjusted for age and gender factors. Model 2: Model 1 + racial background, education level, marital status, PIR, BMI, alcohol consumption frequency, HEI2020, physical activity, hypertension, diabetes, cardiovascular disease, malignant tumors, total cholesterol level, alanine aminotransferase level, aspartate aminotransferase level, glycated hemoglobin, blood glucose level, white blood cell count, and neutrophil count. Model 3: Model 2 + pack-years of smoking, smoking duration, age at smoking initiation, and daily cigarette consumption. TTFC: time to smoke first cigarette after waking.

### Associations between different smoking statuses and cardiovascular disease mortality

Model construction followed the Methods criteria. In Model 1, former smokers showed a higher AHR than non-smokers (AHR=1.11; 95% CI: 0.96–1.28). Among active smokers, TTFC exhibited a U-shaped association with cardiovascular mortality (Table 3).

**Table 3:** Hazard ratios for the association between smoking status and risk of cardiovascular disease (CVD) mortality among US adults, National Health and Nutrition Examination Survey (NHANES), United States, 2001–2018 (N=39084)

*Smoking status*	*Model 1*			*Model 2*			*Model 3*		
	*AHR*	*95% CI*	*p*	*AHR*	*95% CI*	*p*	*AHR*	*95% CI*	*p*
**Non-smoker**	1	-	-	1	-	-	1	-	-
**Former smoker**	1.11	0.96–1.28	0.170	1.03	0.89–1.20	0.710	3.74	1.47–9.48	<0.006
**Smoker TTFC** (min)									
<30	2.72	0.37–2.20	<0.001	1.73	1.37–2.19	<0.001	5.53	2.32–13.14	<0.001
30–60	1.29	0.82–1.93	0.220	1.03	0.69–1.53	0.220	3.45	1.30–9.12	0.010
>60	1.92	1.16–3.18	<0.010	1.55	1.01–2.39	0.010	5.31	2.10–13.44	<0.001

AHR: adjusted hazard ratio. Model 1: adjusted for age and gender factors. Model 2: Model 1 + racial background, education level, marital status, PIR, BMI, alcohol consumption frequency, HEI2020, physical activity, hypertension, diabetes, cardiovascular disease, malignant tumors, total cholesterol level, alanine aminotransferase level, aspartate aminotransferase level, glycated hemoglobin, blood glucose level, white blood cell count, and neutrophil count. Model 3: Model 2 + pack-years of smoking, smoking duration, age at smoking initiation, and daily cigarette consumption. TTFC: time to smoke first cigarette after waking. CVD: cardiovascular disease.

### Associations between different smoking statuses and cancer mortality

**Figure 2:**
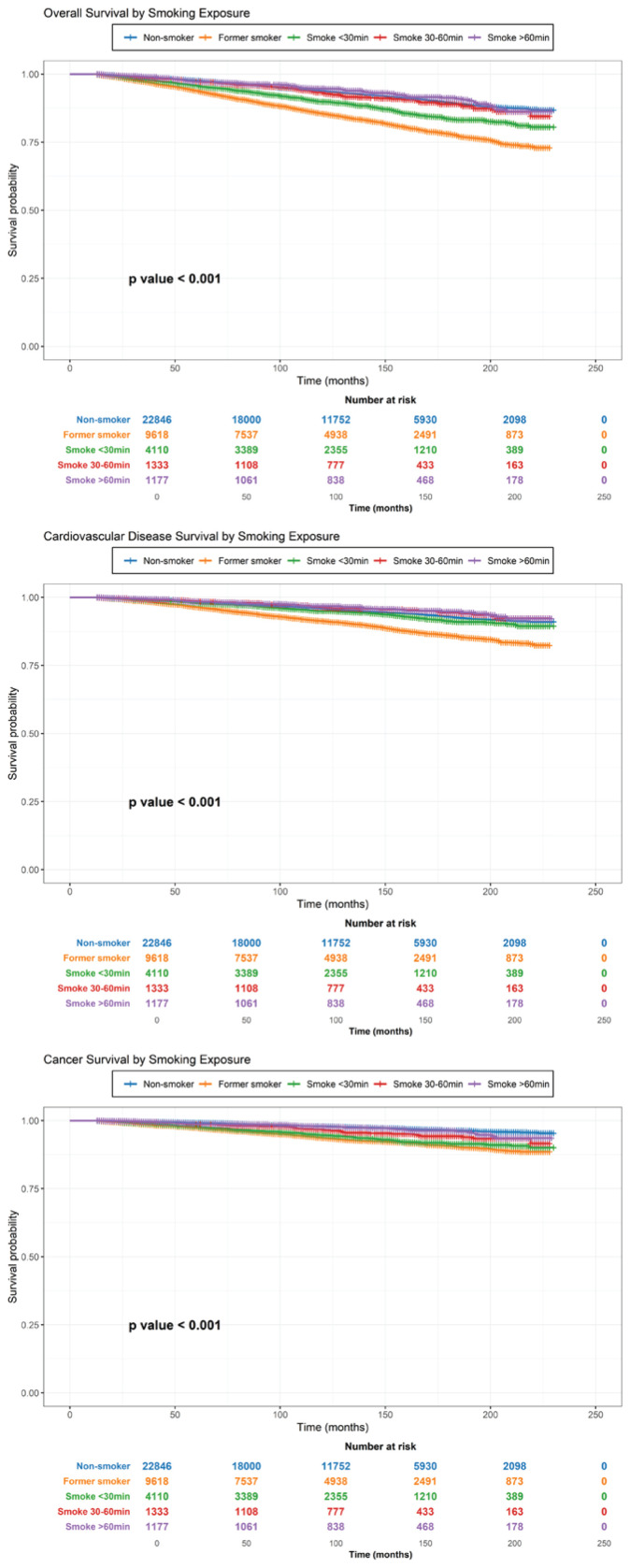
Kaplan-Meier survival curves for all-cause, cardiovascular disease (CVD), and cancer mortality among US adults based on smoking behavior, National Health and Nutrition Examination Survey (NHANES), 2001–2018 (N=39084)

In Model 1, former smokers showed a higher risk of cancer mortality than non-smokers (AHR=1.65; 95% CI: 1.36–2.01, p<0.001). A dose–response relationship was observed between TTFC and cancer mortality, with AHR of 4.49 (95% CI: 3.49–5.78; p<0.001) for TTFC <30 min, 2.39 (95% CI: 1.59–3.59; p<0.001) for 30–60 min, and 2.07 (95% CI: 1.28–3.36; p=0.003) for >60 min (Table 4 and Figure 4). A Kaplan–Meier analysis, using the unadjusted model, supported these findings (Figure 2).

**Table 4:** Hazard ratios for the association between smoking status and risk of cancer mortality among US adults, National Health and Nutrition Examination Survey (NHANES), United States, 2001–2018 (N=39084)

*Smoking status*	*Model 1*			*Model 2*			*Model 3*		
	*AHR*	*95% CI*	*p*	*AHR*	*95% CI*	*p*	*AHR*	*95% CI*	*p*
**Non-smoker**	1	-	-	1	-	-	1	-	-
**Former smoker**	1.65	1.36–2.01	<0.001	1.58	1.29–1.93	<0.001	1.47	0.58–3.72	0.420
**Smoker TTFC** (min)									
<30	4.49	3.49–5.78	<0.001	3.66	2.82–4.76	<0.001	3.23	1.25–8.31	0.020
30–60	2.39	1.59–3.59	<0.001	2.10	1.42–3.10	<0.001	2.03	0.83–5.01	0.120
>60	2.07	1.28–3.36	0.003	1.79	1.10–2.92	0.020	1.82	0.70–4.70	0.220

AHR: adjusted hazard ratio. Model 1: adjusted for age and gender factors. Model 2: Model 1 + racial background, education level, marital status, PIR, BMI, alcohol consumption frequency, HEI2020, physical activity, hypertension, diabetes, cardiovascular disease, malignant tumors, total cholesterol level, alanine aminotransferase level, aspartate aminotransferase level, glycated hemoglobin, blood glucose level, white blood cell count, and neutrophil count. Model 3: Model 2 + pack-years of smoking, smoking duration, age at smoking initiation, and daily cigarette consumption. TTFC: time to smoke first cigarette after waking.

**Figure 3:**
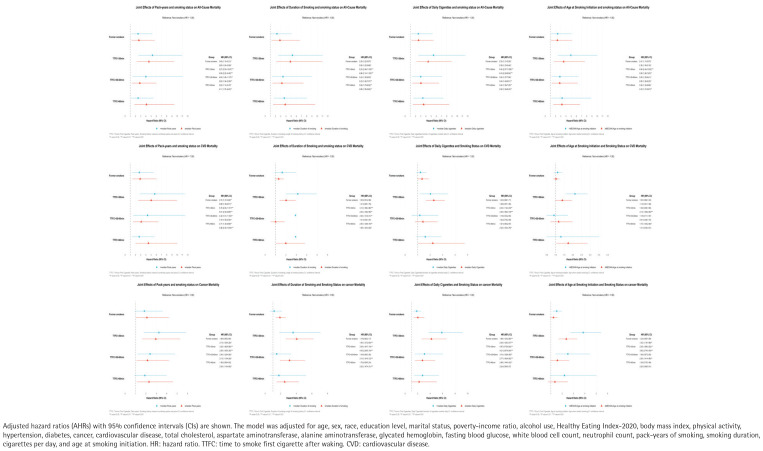
Joint analysis of smoking status and smoking related variables with all-cause, cardiovascular and cancer mortality among US adults, National Health and Nutrition Examination Survey (NHANES), 2001–2018 (N=39084)

**Figure 4:**
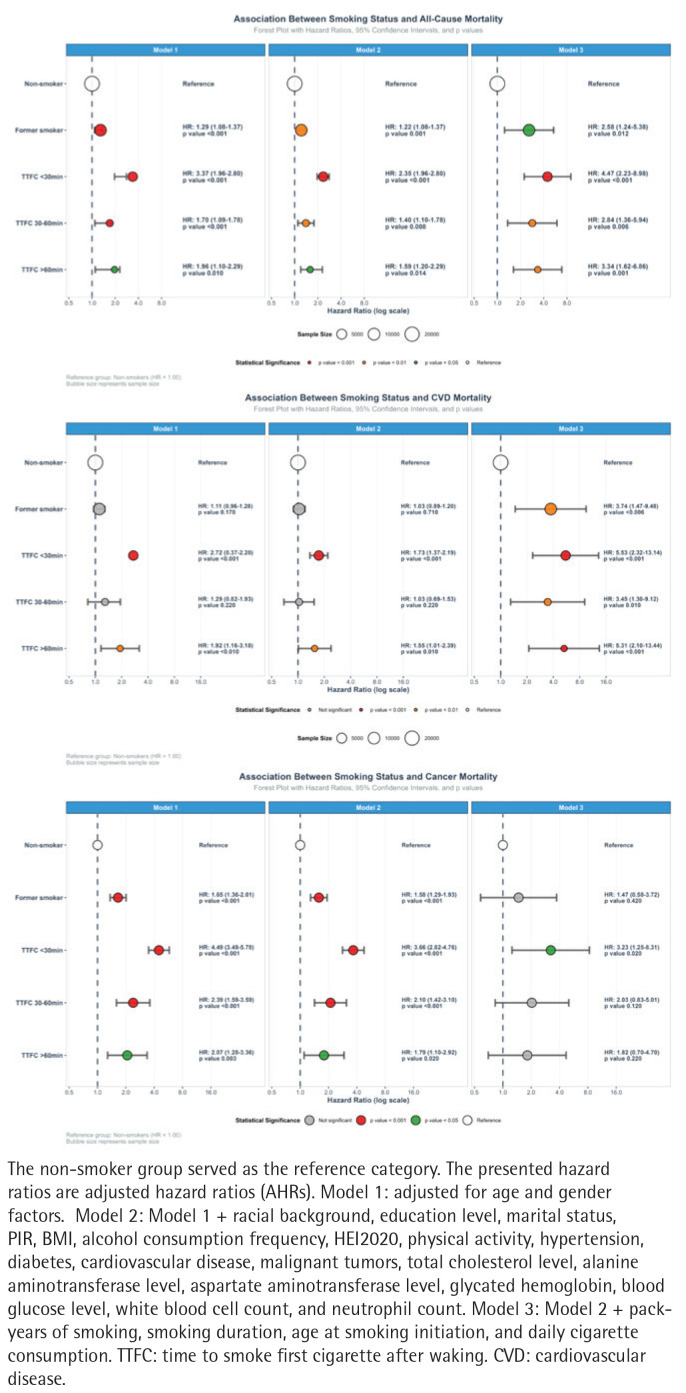
Association between smoking status and mortality among US adults, National Health and Nutrition Examination Survey (NHANES), 2001–2018 (N=39084)

### Competing risk analysis

In addition, to address the competing risks among different causes of death, we further conducted a Fine-Gray competing risk analysis. This analysis confirmed that the dose-response relationship between early-morning smoking and mortality remained stable after accounting for competing risks (Supplementary file Figure 1).

### Subgroup analyses

The association between smoking status and both all-cause and cause-specific mortality was stronger among participants aged ≥60 years. Notably the U-shaped relationship between TTFC and all-cause mortality remained unaffected by age. Furthermore, the association between smoking status and mortality risk was most pronounced among non-Hispanic White participants and least evident among Other Hispanic groups, including Mexican Americans (Supplementary file Tables 2–4). No significant interactions were observed between smoking status and other traditional risk factors.

### Joint analyses between smoking status and smoking-related variables and the risk of all-cause and idiosyncratic mortality

Joint analysis revealed that lower pack-years, shorter smoking duration, fewer cigarettes/day, and shorter TTFC were all associated with higher all-cause mortality. Cardiovascular mortality was linked to greater daily cigarette use, while cancer mortality correlated with longer smoking duration. Earlier smoking initiation and shorter TTFC raised risks across all mortality outcomes (Figure 3).

### Sensitivity analyses

Sensitivity analyses, which involved excluding participants who died within two years of follow-up and those with missing covariate data, yielded results consistent with the primary analyses (Supplementary file Tables 5–10).

## DISCUSSION

This study found that both former and current smokers had higher all-cause and cause-specific mortality than never smokers. TTFC exhibited a U-shaped association with all-cause and cardiovascular mortality, contrasting with the conventional belief that delayed smoking confers protection^17^. These robust findings were consistent across sensitivity and subgroup analyses. Studies from Australia and the United States indicate that former smokers have significantly lower mortality risk than continuous smokers. Current smoking is associated with markedly increased mortality, with smokers losing at least ten years of life expectancy compared to never smokers. Substantial evidence indicates that smoking cessation before the age of 40 years averts the vast majority of the excess mortality risk associated with continued smoking^18,19^. However, our study specifically identified a group for whom short-term smoking cessation remains challenging and employed the TTFC after waking up as a behavioral metric of nicotine dependence, thereby offering substantial clinical relevance^20^.

Our research suggests a paradoxical link between longer TTFC and increased all-cause and cardiovascular mortality. Nicotine dependence involves genetic and social factors, such as ethnicity. While previous TTFC studies largely involved non-US cohorts (e.g. Asian, European, Australian), this study focused on a US population^21,22^. Differences in genetic background and sociocultural context may contribute to heterogeneity in TTFC–health outcome associations across ethnic groups^23^. Second, the sample sizes of the TTFC 30–60 min and TTFC >60 min groups were small in this study^24^. An American study suggests that once tobacco users reach higher stages of nicotine dependence, they often transition quickly to more advanced stages of dependence^25^. Additionally, subgroup analyses revealed that the 30–60 min TTFC group was predominantly female, White, more highly educated, of normal weight, physically active, with fewer heavy drinkers, and with lower levels of total cholesterol, liver transaminases, and blood glucose. These findings are highly consistent with those of previous studies that found that men had lower smoking cessation self-efficacy, smoked more, and were more likely to compensate for smoking^26^. Whites were more likely to attempt unassisted abrupt smoking discontinuation, while a higher level of education was consistently associated with a greater likelihood of immediate cessation^27^. Light smokers were more likely to have a normal BMI than heavy smokers because of fewer weight changes induced by smoking or cessation^28^. Delayed morning smoking often reflects poorer health and socioeconomic stress, with vulnerable groups using smoking to cope. TTFC may not be a straightforward risk marker but is intertwined with complex behavioral patterns^29^. Smoking prevalence decreases during work hours but rises after work and peaks at night, suggesting some delay morning smoking due to work constraints but compensate with increased smoking later^30^. Smokers tend to consume consistent amounts of nicotine daily to achieve the desired effect by adjusting their smoking behavior to regulate nicotine levels^31^.

Smoking within 30 minutes of waking elevates cardiovascular risk during the morning peak incidence period^32^. While delayed smokers may avoid this acute risk window, they still incur long-term cardiovascular damage, particularly through increased smoking frequency or deeper inhalation^33,34^. Nicotine clearance rates exhibit substantial interindividual variation, principally governed by the cytochrome P450 2A6 enzyme. Slower nicotine metabolizers may not need to smoke immediately in the morning to maintain blood levels, but this does not imply lower nicotine toxicity^35^. Instead, they may experience a greater cardiovascular burden due to prolonged nicotine presence in the body, leading to persistent vasoconstriction and sympathetic activation. These findings have significant clinical implications. Specifically, using TTFC >60 min for screening can identify high-risk individuals with compensatory smoking, enabling targeted interventions. Future research is necessary to further validate the utility of TTFC in clinical risk stratification and to further elucidate the behavioral mechanisms underlying compensatory smoking.

### Limitations

This study has several limitations. First, its observational nature precludes causal inference. Despite extensive adjustment for confounders, residual confounding (e.g. mental health, substance use) may persist^36^. At the same time, although smoking behavior usually begins before fatal events and we have excluded samples that died within two years of follow-up in the sensitivity analysis, we cannot completely rule out the possibility of reverse causation, that is, some undiagnosed early diseases may have influenced participants’ reporting of smoking behavior. Secondly, self-reported smoking behavior and TTFC data are susceptible to information bias, such as recall bias, social desirability bias, and even misclassification. Such information biases may weaken the observed effect size, thereby underestimating the true association. Third, TTFC was analyzed categorically, preventing precise dose–response characterization or inflection point identification for the observed U-shaped relationships. Fourth, a lack of data on compensatory smoking (e.g. intensity) limited mechanistic exploration. Fifth, caution is needed when generalizing the results. This study is based on the nationally representative data from the United States, making the findings well-representative of the US population. However, due to significant differences in tobacco policies, cultural practices, and healthcare systems across countries or regions, the applicability of these conclusions to other countries still needs further verification. Finally, varying follow-up durations may affect mortality estimates.

## CONCLUSIONS

Our analyses, based on the well-established but imperfect probabilistic linkage to the National Death Index, confirm elevated mortality risks among both former and current smokers and reveal a U-shaped relationship between TTFC and both all-cause and cardiovascular mortality, but not for cancer mortality. Any misclassification of mortality status from the linkage process would likely lead to an underestimation of the reported associations.

## Supplementary Material



## Data Availability

The data supporting this research are available from the following source: CDC website (https://wwwn.cdc.gov/nchs/nhanes/Default.aspx).
