# Supplementary material for: Association of smoking behavior and time to first cigarette with all-cause and cause-specific mortality: A cohort analysis from the NHANES 2001–2018
Source: Tob Induc Dis. 2026 Feb 19;24:10.18332/tid/215944. doi: 10.18332/tid/215944 (PMC12937990; doi:10.18332/tid/215944)
Supplement: Supplementary file 1 [file TID-24-27-s1.pdf]

Supplementary Table 1. Detailed Definitions of Smoking Status and Time to First Cigarette (TTFC) for Cohort Construction, Based on NHANES Questionnaire Items (2001-2018),United States, (N=39,084)

Supplementary Table 2. Hazard Ratios from Subgroup Analysis for the Association of Smoking Status with All-Cause Mortality, Stratified by Sociodemographic, Lifestyle, and Clinical Factors, National Health and Nutrition Examination Survey (NHANES), United States, 2001-2018 (N=39,084)

Supplementary Table 3. Hazard Ratios from Subgroup Analysis for the Association of Smoking Status with Cardiovascular Disease Mortality, Stratified by Sociodemographic, Lifestyle, and Clinical Factors, National Health and Nutrition Examination Survey (NHANES), United States, 2001-2018 (N=39,084)

Supplementary Table 4. Hazard Ratios from Subgroup Analysis for the Association of Smoking Status with Cancer Mortality, Stratified by Sociodemographic, Lifestyle, and Clinical Factors, National Health and Nutrition Examination Survey (NHANES), United States, 2001-2018 (N=39,084)

Supplementary Table 5. Hazard Ratios and 95% Confidence Intervals for the association between smoking status and All-Cause Mortality after excluding participants who died within two years of follow-up, National Health and Nutrition Examination Survey (NHANES), United States, 2001-2018 (N=36,554)

Supplementary Table 6. Hazard Ratios and 95% Confidence Intervals for the association between smoking status and Cardiovascular disease Mortality after excluding participants who died within two years of follow-up, National Health and Nutrition Examination Survey (NHANES), United States, 2001-2018 (N=36,554)

Supplementary Table 7. Hazard Ratios and 95% Confidence Intervals for the association between smoking status and Cancer Mortality after excluding participants who died within two years of follow-up, National Health and Nutrition Examination Survey (NHANES), United States, 2001-2018 (N=36,554)

Supplementary Table 8. Hazard Ratios and 95% Confidence Intervals for the Association Between Smoking Status and All-Cause Mortality After Excluding Participants with Missing Data, National Health and Nutrition Examination Survey (NHANES), United States, 2001-2018 (N=23,929)

Supplementary Table 9. Hazard Ratios and 95% Confidence Intervals for the Association Between Smoking Status and Cardiovascular Disease Mortality After Excluding Participants with Missing Data, National Health and Nutrition Examination Survey (NHANES), United States, 2001–2018 (N=23,929)

Supplementary Table 10. Hazard Ratios and 95% Confidence Intervals for the Association Between Smoking Status and Cancer Mortality After Excluding Participants with Missing Data, National Health and Nutrition Examination Survey (NHANES), United States, 2001-2018 (N=23,929)

Supplementary Figure 1. Associations of Smoking Status and TTFC with Cause-Specific Mortality Using Fine-Gray Competing Risk Models in a Nationally Representative Cohort of U.S. Adults (NHANES 2001-2018, N=39,084).

Supplementary Table 1. Detailed Definitions of Smoking Status and Time to First Cigarette (TTFC) for Cohort Construction, Based on NHANES Questionnaire Items (2001-2018)

	**Case(n)**	**SMQ**
**Non smker**	22846	SMQ020<100
**Former smoker**	9618	SMQ020>100ANDSMQ040=3
**TTFC<30min**	4110	SMQ020>100ANDSMQ040=1OR2<br>SMQ077/SMQ081<30
**TTFC30-60min**	1333	SMQ020>100ANDSMQ040=1OR2<br>SMQ077/SMQ081 30-60
**TTFC>60min**	1177	SMQ020>100ANDSMQ040=1OR2<br>SMQ077/SMQ081>60

Abbreviations: TTFC, time to first cigarette after waking; SMQ, Smoking Questionnaire (referring to relevant NHANES questionnaire codes: SMQ020, SMQ040, SMQ077, SMQ081).

Supplementary Table 2. Hazard Ratios from Subgroup Analysis for the Association of Smoking Status with All-Cause Mortality, Stratified by Sociodemographic, Lifestyle, and Clinical Factors, National Health and Nutrition Examination Survey (NHANES), United States, 2001-2018 (N=39,084)

		HR (95% CI) P value					
	**Smoking status**	**Non smoker**	**Former smoker**	**TTFC<30min**	**TTFC30-60min**	**TTFC>60min**	**P for interaction**
**Age**	**<60 years**	1.00(reference)	1.15(0.25,5.19)0.86	2.26(0.51,10.05)0.28	1.54(0.32,7.52)0.59	1.91(0.42,8.72)0.41	P<0.01
	**≥60 years**	1.00(reference)	3.8(1.23,11.75)0.02	6.19(2.03,18.89)P<0.01	3.58(1.21,10.56)0.02	4.35(1.42,13.3)0.01	
**Sex**	**Male**	1.00(reference)	2.98(1.23,7.24)0.02	4.59(1.96,10.74)P<0.01	2.32(0.7,7.67)0.17	3.34(1,11.22)0.05	P=0.84
	**Female**	1.00(reference)	2.06(0.64,6.67)0.23	4.1(1.22,13.76)0.02	3.17(1.28,7.9)0.01	3.02(1.18,7.73)0.02	
	**Mexican Americans**	1.00(reference)	2.02(0.25,48.28)0.66	5.23(0.28,98.83)0.27	1.49(0.12,18.96)0.76	3.49(0.22,56.56)0.38	P<0.01

**Race**	**Non-Hispanic Whites**	1.00(reference)	3.69(1.5,9.07)P<0.01	6.61(2.77,15.78)P<0.01	3.51(1.43,8.62)P<0.01	5.15(1.94,13.64)P<0.01	
	**Non-Hispanic Blacks**	1.00(reference)	3.08(0.71,13.37)0.13	4.37(1.01,18.9)0.05	4.22(0.95,18.82)0.06	3.77(0.92,15.5)0.07	
	**Other Hispanics**	1.00(reference)	1.25(0.07,23.91)0.88	2.38(0.13,44.44)0.56	2.87(0.21,39.68)0.43	0.17(0.01,3.73)0.26	
	**Other Races**	1.00(reference)	20.69(0.92,465.6)0.06	26.04(1.04,751.71)0.05	44.07(2.09,929.84)0.02	6.39(0.27,153.32)0.25	
**PIR**	**Low PIR(<1.3)**	1.00(reference)	1.89(0.73,4.87)0.19	3.05(0.51,10.05)0.01	2.41(1.03,5.56)0.07	2.52(1.11,5.69)0.03	P=0.46
	**Middle PIR(1.3-3.5)**	1.00(reference)	2.33(0.69,7.8)0.17	3.64(1.05,12.53)0.04	3.11(0.89,10.86)0.08	2.64(0.66,10.52)0.17	
	**High PIR(≥3.5)**	1.00(reference)	2.90(0.65,12.98)0.16	6.35(1.44,21.06)0.01	1.95(0.41,9.18)0.40	5.6(1.18,7.73)0.03	
**Educatio**	**Less than high school**	1.00(reference)	1.80(0.5,6.46)0.37	3.45(1,11.86)0.05	2.13(0.57,7.94)0.26	2.31(0.64,8.41)0.20	P=0.05

**n level**	**high school or GED**	1.00(reference)	1.49(0.35,6.31)0.59	1.76(0.43,7.29)0.43	1.59(0.37,6.8)0.53	2.41(0.51,11.46)0.27	
	**Above high school**	1.00(reference)	3.78(1.22,11.68)0.02	7.42(2.53,21.74)P<0.01	3.91(1.25,12.23)0.02	4.07(1.28,12.96)0.02	
**Alcohol intake**	**Light drinking**	1.00(reference)	5.10(2.16,12.07)P<0.01	9.01(3.94,20.59)P<0.01	4.43(1.86,10.57)P<0.01	6.05(2.6,14.07)P<0.01	P=0.38
	**Moderate drinking**	1.00(reference)	0.38(0.04,3.74)0.40	0.55(0.07,4.63)0.59	0.75(0.08,6.72)0.80	0.38(0.03,4.76)0.45	
	**Heavy drinking**	1.00(reference)	1.64(0.36,7.46)0.52	2.70(0.59,12.41)0.20	2.17(0.39,12.25)0.38	3.16(0.55,18.04)0.20	
**HEI2020**	**HEI2020 <Median**	1.00(reference)	2.03(0.78,5.29)0.15	3.63(1.45,9.14)P<0.01	2.46(0.9,6.75)0.08	2.86(1.03,8.00)0.05	P=0.10
	**HEI2020 >Median**	1.00(reference)	3.30(1,19,9.18)0.02	5.60(2.07,15.13)P<0.01	3.02(1.31,8.06)0.03	3.84(1.27,11.57)0.02	
**Physical activity**	**Light physical activity**	1.00(reference)	2.95(1,8.68)0.05	5.24(1.84,14.87)P<0.01	3.90(1.21,12.62)0.02	6.03(1.81,20.06)P<0.01	P=0.14
	**Moderate physical activity**	1.00(reference)	2.70(0.98,7.42)0.06	4.43(1.67,11.76)P<0.01	2.36(0.94,5.95)0.07	2.77(1.07,7.2)0.04	

	**Vigorous physical activity**	1.00(reference)	3.82(0.04,335.1)0.56	9.05(0.12,709.9)0.32	18.73(0.27,1292)0.18	1.80(0.02,203)0.81	
**Hypertension**	**Hypertension**	1.00(reference)	5.60(1.91,16.41)P<0.01	8.87(3.15,25.02)P<0.01	6.26(2.12,18.49)P<0.01	6.99(2.25,21.69)P<0.01	P=0.93
	**Non Hypertension**	1.00(reference)	1.27(0.39,4.13)0.70	2.42(0.82,7.14)0.11	1.31(0.37,4.72)0.68	1.77(0.60,5.17)0.30	
**Diabetes**	**Diabetes**	1.00(reference)	3.44(0.81,14.66)0.10	4.59(1.17,18.09)0.03	4.12(0.92,18.5)0.07	1.57(0.34,7.31)0.57	P=0.10
	**Non Diabetes**	1.00(reference)	2.58(1.08,6.2)0.03	4.71(2.01,11.04)P<0.01	2.91(1.2,7.06)0.02	4.47(1.88,10.58)P<0.01	
**Total Cholesterol**	**Total Cholesterol <Median**	1.00(reference)	2.17(0.89,5.26)0.09	3.85(1.61,9.23)P<0.01	2.26(0.93,5.48)0.07	1.83(0.62,5.46)0.28	P=0.46
	**Total Cholesterol >Median**	1.00(reference)	2.92(1.02.8.36)0.05	4.94(1.74,14.04)P<0.01	3.39(1.06,10.8)0.04	4.95(1.82,13.44)P<0.01	

Note: The non-smoker group served as the reference category. The presented hazard ratios are adjusted hazard ratios (AHRs).

Confounding factor: The model has been adjusted for age, sex, race, education level, marital status, PIR, BMI, alcohol consumption frequency, HEI2020, physical activity, hypertension, diabetes, cardiovascular disease and malignant tumors, total cholesterol, alanine aminotransferase, aspartate aminotransferase, glycated hemoglobin, blood glucose, white blood cell count, neutrophil count, pack-years of smoking, smoking

duration, age at smoking initiation, and daily cigarette consumption.(And make sure to exclude the stratified variables from the model each time.)
Abbreviations: HR, hazard ratio; CI, confidence interval; TTFC, time to first cigarette after waking; P, P value;PIR, poverty-income ratio; GED, General Educational Development (high school equivalency); BMI, body mass index; CVD, cardiovascular disease; HEI2020, Healthy Eating Index-2020.

Supplementary Table 3. Hazard Ratios from Subgroup Analysis for the Association of Smoking Status with Cardiovascular Diseases Mortality, Stratified by Sociodemographic, Lifestyle, and Clinical Factors, National Health and Nutrition Examination Survey (NHANES), United States,

2001-2018 (N=39,084)

		HR (95% CI) P value					
	**Smoking status**	**Non smoker**	**Former smoker**	**TTFC<30min**	**TTFC30-60min**	**TTFC>60min**	**P for interaction**
**Age**	**<60 years**	1.00(reference)	2.49(0.43,14.50)0.31	4.26(0.81,22.44)0.09	3.41(0.53,21.87)0.20	4.89(0.84,28.54)0.08	P<0.01
	**≥60 years**	1.00(reference)	2.73(0.61,12.16)0.19	4.17(0.93,18.74)0.06	2.14(0.54,8.47)0.28	3.37(0.80,14.15)0.10	
**Sex**	**Male**	1.00(reference)	2.54(0.68,9.42)0.16	3.85(1.07,13.76)0.04	2.56(062,10.57)0.19	4.14(1.05,16.39)0.04	P=0.92
	**Female**	1.00(reference)	4.19(0.84,20.84)0.08	6.64(1.39,29.93)0.02	3.87(0.71,21.02)0.12	5.28(1.06,26.35)0.04	
**Race**	**Mexican Americans**	1.00(reference)	3.36(0.02,706.83) 0.66	9.27(0.06,1340.00)0.38	1.88(0.01,277.70) 0.80	5.08(0.06,465.94)0.48	P<0.01
	**Non-Hispanic Whites**	1.00(reference)	7.02(2.17,22.68)P<0.01	11.10(3.54,34.84) P<0.01	5.03(1.4317.70)0.01	12.32(3.43,44.24)P<0.01	
	**Non-Hispanic Blacks**	1.00(reference)	3.25(0.57,18.68)0.19	5.15(0.86,30.89)0.07	4.53(0.65,31.68)0.13	5.01(0.92,27.23)0.06	
	**Other Hispanics**	1.00(reference)	3.04(0.07,131.40) 0.56	5.68(0.10,322.10) 0.40	6.39(0.20,202.60) 0.29	1.086e-7(8.2e-10,1.4e-5)P<0.01	
	**Other Races**	1.00(reference)	9.01(0.14,577.86) 0.30	3.42(0.03,342.09) 0.60	37.17(0.40,3457.02)0.12	6.93(0.07,650.46)0.40	

**PIR**	**Low PIR(<1.3)**	1.00(reference)	1.89(0.73,4.87)0.19	3.05(0.51,10.05)0.01	2.41(1.03,5.56)0.07	2.52(1.11,5.69)0.03	P=0.46
	**Middle PIR(1.3-3.5)**	1.00(reference)	2.33(0.69,7.8)0.17	3.64(1.05,12.53)0.04	3.11(0.89,10.86)0.08	2.64(0.66,10.52)0.17	
	**High PIR(≥ 3.5)**	1.00(reference)	2.90(0.65,12.98)0.16	6.35(1.44,21.06)0.01	1.95(0.41,9.18)0.40	5.6(1.18,7.73)0.03	
**Education level**	**Less than high school**	1.00(reference)	1.93(0.30,12.46)0.49	2.79(0.50,15.48)0.24	2.34(0.36,15.24)0.37	2.42(0.42,14.03)0.33	P=0.05
	**high school or GED**	1.00(reference)	4.17(0.65,26.90)0.13	4.83(0.71,32.89)0.11	5.08(0.74,34.62)0.10	9.59(1.34,68.40)0.02	
	**Above high school**	1.00(reference)	6.53(1.06,40.34)0.04	13.08(2.18,78.35)P<0.01	4.03(0.62,26.18)0.15	8.29(1.24,55.44)0.03	
**Alcohol intake**	**Light drinking**	1.00(reference)	6.13(1.80,20.85)P<0.01	9.36(2.85,30.73)P<0.01	4.61(1.34,15.82)0.02	8.47(2.46,29.12)P<0.01	P=0.02
	**Moderate drinking**	1.00(reference)	0.95(0.03,26.25)0.98	1.32(0.08,23.09)0.85	2.02(0.07,60.13)0.69	0.34(0.01,8.24)0.51	
	**Heavy drinking**	1.00(reference)	2.15(0.19,24.08)0.53	3.38(0.27,42.64)0.35	2.23(0.16,31.17)0.55	7.30(0.46,16.24)0.16	
**HEI2020**	**HEI2020<Median**	1.00(reference)	3.80(1.21,11.94)0.02	6.39(2.23,18.27)P<0.01	4.74(1.45,15.45)0.01	6.04(1.79,20.39)P<0.01	P=0.09
	**HEI2020>Median**	1.00(reference)	3.69(0,76,18.01)0.11	4.57(0.99,21.10)0.05	1.93(0.34,10.91)0.46	4.66(0.85,25.64)0.08	
	**Light physical**	1.00(reference)	7.04(1.71,28.92)P<0.01	10.56(2.74,40.62)P<0.01	7.16(1.41,36.52)0.02	14.68(3.20,67.37)P<0.01	P=0.07

	**activity**						
**Physical activity**	**Moderate physical activity**	1.00(reference)	3.32(0.89,12.38)0.07	4.76(1.32,17.16)0.02	2.92(0.78,10.88)0.11	4.04(1.15,14.25)0.03	
	**Vigorous physical activity**	1.00(reference)	3.01(0.00,1891.77)0.74	11.15(0.01,8497.53)0.48	16.15(0.03,9568.86)0.39	4.08(0.01,2128.6)0.66	
**Hypertension**	**Hypertension**	1.00(reference)	10.21(2.66,39.20) P<0.01	13.63(3.53,52.67) P<0.01	8.88(2.13,36.93)P<0.01	13.57(2.99,61.50)P<0.01	P=1.00
	**Non Hypertension**	1.00(reference)	1.19(0.16,8.78)0.86	2.35(0.39,14.08)0.35	1.49(0.17,13.08)0.72	2.21(0.34,14.21)0.40	
**Diabetes**	**Diabetes**	1.00(reference)	5.25(0.99,27.94)0.05	6.83(1.37,33.94)0.02	4.03(0.65,24.89)0.13	3.00(0.53,16.88)0.21	P=0.16
	**Non Diabetes**	1.00(reference)	4.03(1.20,13.54)0.02	6.45(2.04,20.41)P<0.01	4.72(1.36,16.38)0.01	9.18(2.70,31.27)P<0.01	
**Total Cholesterol**	**Total Cholesterol <Median**	1.00(reference)	3.09(0.72,13.34)0.13	5.62(1.37,23.07)0.02	2.03(0.46,8.96)0.35	3.64(0.65,20.48)0.14	P=0.32
	**Total Cholesterol >Median**	1.00(reference)	5.10(1.51.17.18)P<0.01	6.78(2.09,22.04)P<0.01	6.89(1.76,26.99)P<0.01	8.92(2.92,27.23)P<0.01	

Note: The non-smoker group served as the reference category. The presented hazard ratios are adjusted hazard ratios (AHRs).

Confounding factor: The model has been adjusted for age, sex, race, education level, marital status, PIR, BMI, alcohol consumption frequency, HEI2020, physical activity, hypertension, diabetes, cardiovascular disease and malignant tumors, total cholesterol, alanine aminotransferase, aspartate aminotransferase, glycated hemoglobin, blood glucose, white blood cell count, neutrophil count, pack-years of smoking, smoking duration, age at smoking initiation, and daily cigarette consumption.(And make sure to exclude the stratified variables from the model each time.)

Abbreviations: HR, hazard ratio; CI, confidence interval; TTFC, time to first cigarette after waking; P, P value;PIR, poverty-income ratio; GED, General Educational Development (high school equivalency); BMI, body mass index; CVD, cardiovascular disease; HEI2020, Healthy Eating Index-2020.

Supplementary Table 4. Hazard Ratios from Subgroup Analysis for the Association of Smoking Status with Cancer Mortality, Stratified by Sociodemographic, Lifestyle, and Clinical Factors, National Health and Nutrition Examination Survey (NHANES), United States, 2001-2018 (N=39,084)

		HR (95% CI) P value					
	**Smoking status**	**Non smoker**	**Former smoker**	**TTFC<30min**	**TTFC30-60min**	**TTFC>60min**	**P for interaction**
**Age**	**<60 years**	1.00(reference)	0.34(0.03,3.38)0.36	0.83(0.08,8.93)0.88	0.55(0.05,6.24)0.63	0.50(0.04,6.33)0.59	P=0.95
	**≥60 years**	1.00(reference)	3.42(0.66,17.70)0.14	6.57(1.24,34.72)0.03	4.33(0.82,22.91)0.08	4.30(0.77,23.98)0.10	
**Sex**	**Male**	1.00(reference)	3.35(1.00,11.25)0.05	6.34(1.90,21.19)P<0.01	4.79(1.46,15.73)0.01	1.84(0.49,6.90)0.36	P=0.14
	**Female**	1.00(reference)	0.67(0.15,2.92)0.59	1.94(0.39,9.67)0.42	1.02(0.22,4.82)0.98	1.53(0.31,7.50)0.60	
**Race**	**Mexican Americans**	1.00(reference)	1.57(0.27,9.08)0.61	4.05(0.51,32.54)0.19	1.40(0.21,9.26)0.73	4.24(0.51,35.38)0.18	P<0.01
	**Non-Hispanic Whites**	1.00(reference)	1.37(0.41,4.63)0.61	3.22(0.92,11.25)0.07	2.05(0.62,6.77)0.24	1.79(0.48,6.62)0.38	

	**Non-Hispanic Blacks**	1.00(reference)	2.11(0.38,11.58)0.39	2.80(0.56,14.04)0.21	3.04(0.53,17.31)0.21	2.12(0.33,13.60)0.43	
	**Other Hispanics**	1.00(reference)	0,57(0.01,22.16)0.76	1.07(0.03,34.49)0.97	1.48(0.03,77.05)0.85	0.18(0.01,5.96)0.34	
	**Other Races**	1.00(reference)	61.24(1.00,3.76)0.05	288(4.21,19.69)P<0.01	1.13e-6(2.23e-8,5.72e-5) P<0.01	1.96e-6(4.66e-8,8.27e-5) P<0.01	
**PIR**	**Low PIR(<1.3)**	1.00(reference)	1.89(0.73,4.87)0.19	3.05(0.51,10.05)0.01	2.41(1.03,5.56)0.07	2.52(1.11,5.69)0.03	P=0.46
	**Middle PIR(1.3-3.5)**	1.00(reference)	2.33(0.69,7.8)0.17	3.64(1.05,12.53)0.04	3.11(0.89,10.86)0.08	2.64(0.66,10.52)0.17	
	**High PIR(≥ 3.5)**	1.00(reference)	2.90(0.65,12.98)0.16	6.35(1.44,21.06)0.01	1.95(0.41,9.18)0.40	5.6(1.18,7.73)0.03	
**Education level**	**Less than high school**	1.00(reference)	1.59(0.35,7.23)0.55	4.42(0.99,19.70)0.05	2.08(0.43,10.08)0.37	2.06(0.37,11.39)0.41	P=0.05
	**high school or**	1.00(reference)	0.45(0.07,3.05)0.41	0.58(0.08,4.33)0.59	0.45(0.06,3.37)0.44	0.51(0.07,3.60)0.50	

	**GED**						
	**Above high school**	1.00(reference)	2.60(0.71,9.45)0.15	6.09(1.70,21.82)P<0.01	4.70(1.28,17.29)0.02	3.29(0.70,15.59)0.13	
**Alcohol intake**	**Light drinking**	1.00(reference)	3.40(1.16,9.98)0.03	8.23(2.87,23.60)P<0.01	4.08(1.27,13.10)0.02	3.89(1.25,12.05)0.02	P=0.35
	**Moderate drinking**	1.00(reference)	0.16(0.02,1.66)0.12	0.26(0.03,2.61)0.25	0.52(0.06,4.66)0.56	0.42(0.02,7.24)0.55	
	**Heavy drinking**	1.00(reference)	1.72(0.24,12.27)0.59	3.48(0.48,25.02)0.22	2.95(0.37,23.63)0.31	1.71(0.21,14.15)0.62	
**HEI2020**	**HEI2020<Median**	1.00(reference)	0.77(0.21,2.76)0.68	1.69(0.46,6.21)0.43	1.06(0.28,4.00)0.93	1.05(0.25,4.49)0.95	P=0.26
	**HEI2020>Median**	1.00(reference)	3.07(0.86,10.97)0.08	7.84(2.06,29.80)P<0.01	5.06(1.51,16.94)P<0.01	3.72(1.08,12.77)0.04	
	**Light physical**	1.00(reference)	0.85(0.21,3.50)0.82	2.13(0.48,9.47)0.32	2.15(0.45,10.19)0.34	1.76(0.41,7.59)0.45	P=0.81

	**activity**						
**Physical activity**	**Moderate physical activity**	1.00(reference)	1.77(0.51,6.11)0.37	3.91(1.11,13.69)0.03	1.85(0.55,6.17)0.32	2.00(0.53,764)0.31	
	**Vigorous physical activity**	1.00(reference)	6.81(0.004,9516.24)0.60	12.15(0.02,9216.49)0.46	44.98(0.05,38906.49)0.27	7.47e-7(9.75e-10,72e-4) P<0.01	
**Hypertension**	**Hypertension**	1.00(reference)	2.53(0.56,7.05)0.50	5.66(1.31,24.36)0.02	4.37(1.03,18.56)0.05	3.02(0.66,13.79)0.15	P=0.61
	**Non Hypertension**	1.00(reference)	1.19(0.34,4.14)0.79	2.63(0.75,9.20)0.13	1.39(0.38,5.09)0.62	1.65(0.38,7.05)0.50	
**Diabetes**	**Diabetes**	1.00(reference)	1.27(0.11,14.52)0.85	2.17(0.19,24.50)0.53	3.14(0.24,40.85)0.38	0.49(0.03,8.39)0.63	P=0.10
	**Non Diabet**	1.00(reference)	1.48(0.54,4.07)0.44	3.47(1.22,9.88)0.02	1.99(0.72,5.51)0.18	2.34(0.82,6.67)0.11	

	**es**						
**Total Cholesterol**	**Total Cholesterol <Median**	1.00(reference)	1.18(0.42,3.32)0.75	2.46(0.83,6.21)0.10	2.27(0.83,6.21)0.11	0.93(0.27,3.23)0.91	P=0.27
	**Total Cholesterol >Median**	1.00(reference)	1.71(0.38.7.67)0.48	4.18(0.91,19.33)0.07	1.88(0.40,8.82)0.42	3.14(0.67,14.87)0.15	

Note: The non-smoker group served as the reference category. The presented hazard ratios are adjusted hazard ratios (AHRs).

Confounding factor: The model has been adjusted for age, sex, race, education level, marital status, PIR, BMI, alcohol consumption frequency, HEI2020, physical activity, hypertension, diabetes, cardiovascular disease and malignant tumors, total cholesterol, alanine aminotransferase, aspartate aminotransferase, glycated hemoglobin, blood glucose, white blood cell count, neutrophil count, pack-years of smoking, smoking duration, age at smoking initiation, and daily cigarette consumption.(And make sure to exclude the stratified variables from the model each time.)

Abbreviations: HR, hazard ratio; CI, confidence interval; TTFC, time to first cigarette after waking; P, P value;PIR, poverty-income ratio; GED, General Educational Development (high school equivalency); BMI, body mass index; CVD, cardiovascular disease; HEI2020, Healthy Eating Index-2020.

Supplementary Table 5. Hazard Ratios and 95% Confidence Intervals for the Association Between Smoking Status and All-Cause Mortality After Excluding Participants Who Died Within Two Years of Follow-up (Sensitivity Analysis), National Health and Nutrition Examination Survey (NHANES), United States, 2001-2018 (N=36,554)

All-Cause morality	Model1			Model2			Model3		
	HR	95%CI	P-value	HR	95%CI	P-value	HR	95%CI	P-value
**Non-smoker**	1	-	-	1	-	-	1	-	-
**Former smoker**	1.30	1.16,1.47	<0.001	1.24	1.10,1.39	0.001	2.75	1.83,4.13	0.001
**TTFC<30min**	3.34	2.81,3.99	<0.001	2.38	1.98,2.87	<0.001	4.90	3.36,7.15	<0.001
**TTFC:30-60min**	1.57	1.17,2.09	0.002	1.36	1.03,1.80	0.028	2.98	2.01,4.43	0.001
**TTFC>60min**	1.90	1.28,2.82	0.002	1.57	1.08,2.27	0.017	3.50	2.37,5.18	<0.001

Note: The non-smoker group served as the reference category. The presented hazard ratios are adjusted hazard ratios (AHRs).

Model 1: Adjusted for age and gender factors.

Model 2: Model 1 + racial background, education level, marital status, PIR, BMI, alcohol consumption frequency, HEI2020, physical activity, hypertension, diabetes, cardiovascular disease, malignant tumors, total cholesterol level, alanine aminotransferase level, aspartate aminotransferase level, glycated hemoglobin, blood glucose level, white blood cell count, and neutrophil count.

Model 3: Model 2 + pack-years of smoking, smoking duration, age at smoking initiation, and daily cigarette consumption.

Abbreviations: HR, hazard ratio; CI, confidence interval; TTFC, time to smoke first cigarette after waking.

Supplementary Table 6. Hazard Ratios and 95% Confidence Intervals for the association between smoking status and Cardiovascular disease Mortality after excluding participants who died within two years of follow-up (Sensitivity Analysis), National Health and Nutrition Examination Survey (NHANES), United States, 2001-2018 (N=36,554)

**CVD morality**	**Model1**			**Model2**			**Model3**		
	**HR**	**95%CI**	**P-value**	**HR**	**95%CI**	**P-value**	**HR**	**95%CI**	**P-value**
**Non-smoker**	1	-	-	1	-	-	1	-	-
**Former smoker**	1.12	0.97,1.30	0.135	1.13	0.98,1.31	0.104	3.20	1.58,6.47	0.001
**TTFC<30min**	2.53	2.02,3.16	<0.001	1.82	1.45,2.29	<0.001	4.65	2.38,9.10	<0.001
**TTFC:30-60min**	1.19	0.77,1.83	0.431	1.09	0.71,1.67	0.699	2.91	1.26,6.75	0.013
**TTFC>60min**	1.75	1.06,2.89	0.028	1.49	0.92,2.41	0.101	4.11	1.83,9.23	<0.001

Note: The non-smoker group served as the reference category. The presented hazard ratios are adjusted hazard ratios (AHRs).
Model 1: Adjusted for age and gender factors.
Model 2: Model 1 + racial background, education level, marital status, PIR, BMI, alcohol consumption frequency, HEI2020, physical activity, hypertension, diabetes, cardiovascular disease, malignant tumors, total cholesterol level, alanine aminotransferase level, aspartate aminotransferase level, glycated hemoglobin, blood glucose level, white blood cell count, and neutrophil count.
Model 3: Model 2 + pack-years of smoking, smoking duration, age at smoking initiation, and daily cigarette consumption.
Abbreviations: HR, hazard ratio; CI, confidence interval; TTFC, time to smoke first cigarette after waking; CVD, cardiovascular disease.

Supplementary Table 7. Hazard Ratios and 95% Confidence Intervals for the association between smoking status and Cancer Mortality after excluding participants who died within two years of follow-up (Sensitivity Analysis), National Health and Nutrition Examination Survey (NHANES), United States, 2001-2018 (N=36,554)

Cancer morality	Model1			Model2			Model3		
	**HR**	**95%CI**	**P-value**	**HR**	**95%CI**	**P-value**	**HR**	**95%CI**	**P-value**
**Non-smoker**	1	-	-	1	-	-	1	-	-
**Former smoker**	1.68	1.35,2.09	<0.001	1.61	1.30,2.00	<0.001	1.85	0.79,4.35	0.156
**TTFC<30min**	4.80	3.60,6.40	<0.001	3.92	2.93,5.25	<0.001	4.36	1.83,10.39	<0.001
**TTFC:30-60min**	2.25	1.48,3.34	<0.001	2.03	1.36,3.04	<0.001	2.48	1.08,5.70	0.033
**TTFC>60min**	2.20	1.33,3.63	0.002	1.88	1.13,3.12	0.015	2.33	0.94,5.78	0.067

Note: The non-smoker group served as the reference category. The presented hazard ratios are adjusted hazard ratios (AHRs).
Model 1: Adjusted for age and gender factors.
Model 2: Model 1 + racial background, education level, marital status, PIR, BMI, alcohol consumption frequency, HEI2020, physical activity, hypertension, diabetes, cardiovascular disease, malignant tumors, total cholesterol level, alanine aminotransferase level, aspartate aminotransferase level, glycated hemoglobin, blood glucose level, white blood cell count, and neutrophil count.
Model 3: Model 2 + pack-years of smoking, smoking duration, age at smoking initiation, and daily cigarette consumption.
Abbreviations: HR, hazard ratio; CI, confidence interval; TTFC, time to smoke first cigarette after waking.

Supplementary Table 8. Hazard Ratios and 95% Confidence Intervals for the Association Between Smoking Status and All-Cause Mortality After Excluding Participants with Missing Data (Sensitivity Analysis), National Health and Nutrition Examination Survey (NHANES), United States, 2001-2018 (N=23,929)

**All-Cause morality**	**Model1**			**Model2**			**Model3**		
	**HR**	**95%CI**	**P-value**	**HR**	**95%CI**	**P-value**	**HR**	**95%CI**	**P-value**
**Non-smoker**	1	-	-	1	-	-	1	-	-
**Former smoker**	1.27	1.14,1.41	<0.001	1.14	1.01,1.28	0.035	2.43	1.32,4.46	0.005
**TTFC<30min**	3.25	2.76,3.82	<0.001	1.93	1.54,2.41	<0.001	3.91	2.11,7.23	<0.001
**TTFC:30-60min**	1.64	1.27,2.12	<0.001	1.58	1.16,2.16	0.004	3.37	1.77,6.42	<0.001
**TTFC>60min**	1.97	1.32,2.93	<0.001	1.46	0.96,2.23	0.078	3.12	1.53,6.36	0.002

Note: The non-smoker group served as the reference category. The presented hazard ratios are adjusted hazard ratios (AHRs).
Model 1: Adjusted for age and gender factors.
Model 2: Model 1 + racial background, education level, marital status, PIR, BMI, alcohol consumption frequency, HEI2020, physical activity, hypertension, diabetes, cardiovascular disease, malignant tumors, total cholesterol level, alanine aminotransferase level, aspartate aminotransferase level, glycated hemoglobin, blood glucose level, white blood cell count, and neutrophil count.

Model 3: Model 2 + pack-years of smoking, smoking duration, age at smoking initiation, and daily cigarette consumption.
Abbreviations: HR, hazard ratio; CI, confidence interval; TTFC, time to smoke first cigarette after waking.

Supplementary Table 9. Hazard ratios and 95% confidence intervals for the association between smoking status and cardiovascular disease mortality after excluding participants with missing data (n=23,929).

CVD morality	Model1			Model2			Model3		
	HR	95%CI	P-value	HR	95%CI	P-value	HR	95%CI	P-value
**Non-smoker**	1	-	-	1	-	-	1	-	-
**Former smoker**	1.07	0.93,1.24	0.339	0.96	0.84,1.10	0.591	2.57	1.26,5.25	0.010
**TTFC<30min**	2.64	2.13,3.26	<0.001	1.59	1.19,2.31	0.002	3.94	1.85,8.40	<0.001
**TTFC:30-60min**	1.24	0.84,1.83	0.279	1.19	0.74,1.91	0.478	2.97	1.23,7.19	0.016
**TTFC>60min**	1.86	1.15,3.02	0.012	1.44	0.88,2.38	0.150	3.70	1.55,8.81	0.003

Note: The non-smoker group served as the reference category. The presented hazard ratios are adjusted hazard ratios (AHRs).
Model 1: Adjusted for age and gender factors.
Model 2: Model 1 + racial background, education level, marital status, PIR, BMI, alcohol consumption frequency, HEI2020, physical activity,

hypertension, diabetes, cardiovascular disease, malignant tumors, total cholesterol level, alanine aminotransferase level, aspartate aminotransferase level, glycated hemoglobin, blood glucose level, white blood cell count, and neutrophil count.

Model 3: Model 2 + pack-years of smoking, smoking duration, age at smoking initiation, and daily cigarette consumption.

Abbreviations: HR, hazard ratio; CI, confidence interval; TTFC, time to smoke first cigarette after waking; CVD, cardiovascular disease.

Supplementary Table 10. Hazard Ratios and 95% Confidence Intervals for the Association Between Smoking Status and Cancer Mortality After Excluding Participants with Missing Data (Sensitivity Analysis), National Health and Nutrition Examination Survey (NHANES), United States, 2001-2018 (N=23,929)

**Cancer morality**	**Model1**			**Model2**			**Model3**		
	**HR**	**95%CI**	**P-value**	**HR**	**95%CI**	**P-value**	**HR**	**95%CI**	**P-value**
**Non-smoker**	1	-	-	1	-	-	1	-	-
**Former smoker**	1.64	1.36,1.97	<0.001	1.44	1.17,1.77	<0.001	1.99	0.77,5.17	0.157
**TTFC<30min**	4.32	3.40,5.49	<0.001	2.72	1.99,3.71	<0.001	3.78	1.40,10.22	0.009
**TTFC:30-60min**	2.34	1.59,3.46	<0.001	2.33	1.43,3.79	<0.001	3.55	1.41,8.95	0.007
**TTFC>60min**	2.18	1.37,3.47	0.001	1.62	0.91,2.87	0.099	2.43	0.85,6.95	0.098

Note: The non-smoker group served as the reference category. The presented hazard ratios are adjusted hazard ratios (AHRs).

Model 1: Adjusted for age and gender factors.
Model 2: Model 1 + racial background, education level, marital status, PIR, BMI, alcohol consumption frequency, HEI2020, physical activity, hypertension, diabetes, cardiovascular disease, malignant tumors, total cholesterol level, alanine aminotransferase level, aspartate aminotransferase level, glycated hemoglobin, blood glucose level, white blood cell count, and neutrophil count.
Model 3: Model 2 + pack-years of smoking, smoking duration, age at smoking initiation, and daily cigarette consumption.
Abbreviations: HR, hazard ratio; CI, confidence interval; TTFC, time to smoke first cigarette after waking.

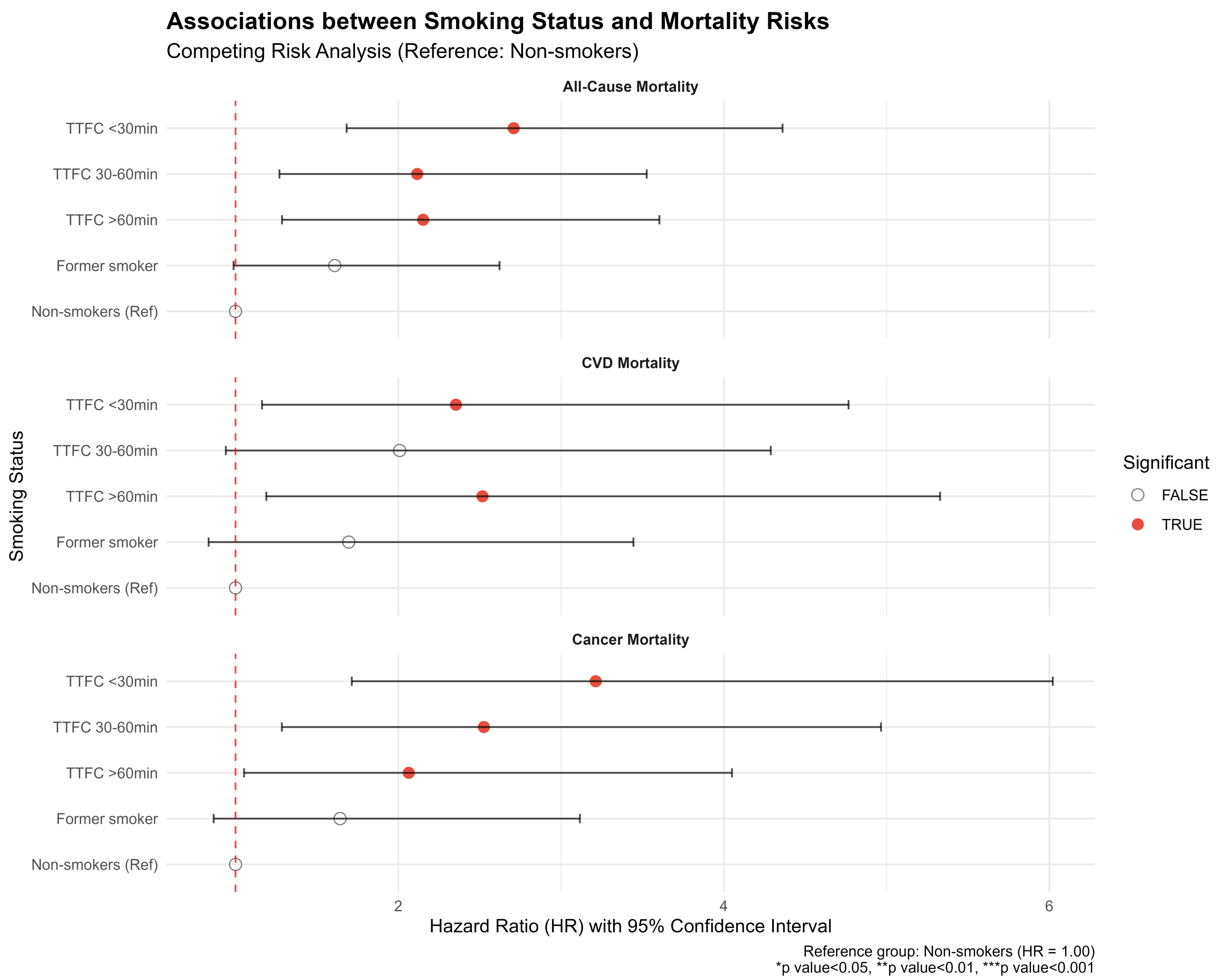

Associations between Smoking Status and Mortality Risks
Competing Risk Analysis (Reference: Non-smokers)
All-Cause Mortality
CVD Mortality
Cancer Mortality
TTFC <30min
TTFC 30-60min
TTFC >60min
Former smoker
Non-smokers (Ref)
Smoking Status
Significant
FALSE
TRUE
2
4
6
Hazard Ratio (HR) with 95% Confidence Interval
Reference group: Non-smokers (HR = 1.00)
*p value<0.05, **p value<0.01, ***p value<0.001

Supplementary Figure 1. Associations of Smoking Status and TTFC with Cause-Specific Mortality Using Fine-Gray Competing Risk Models in a Nationally Representative Cohort of U.S. Adults (NHANES 2001-2018, N=39,084).

Note: Adjusted hazard ratios (AHRs) with 95% confidence intervals (CIs) are shown.

All-cause mortality was analyzed using traditional Cox proportional hazards models, while cause-specific mortality was analyzed using Fine-Gray competing risk models.

The model was adjusted for age, sex, race, education, marital status, poverty-income ratio, alcohol use, Healthy Eating Index-2020, body mass index, physical activity, hypertension, diabetes, cancer, cardiovascular disease, total cholesterol, aspartate aminotransferase, alanine aminotransferase, glycated hemoglobin, fasting blood glucose, white blood cell count, neutrophil count, pack-years of smoking, smoking duration, cigarettes per day, and age at smoking initiation.

HR, hazard ratio; CI, confidence interval; TTFC, time to smoke first cigarette after waking; CVD, cardiovascular disease.

© **2026 Bao N. et al.**
